# The Herniated Heart: A Rare Cause of Myocardial Infarction Identified by Multimodality Imaging

**DOI:** 10.1016/j.case.2025.12.003

**Published:** 2026-03-03

**Authors:** Bria Rice, Samantha Espinosa, Aya Elalfy, Kevin Tayon, Amy Pollak, Pranesh Parikh, Peter Pollak

**Affiliations:** a Department of Cardiovascular Medicine, Mayo Clinic, Jacksonville, Florida; b Department of Cardiovascular Medicine, Brigham and Women’s Hospital, Boston, Massachusetts; c Department of Internal Medicine, Mayo Clinic, Jacksonville, Florida

**Keywords:** Pericardial defect, Myocardial infarction, Acute coronary syndrome, Pericardiectomy

## Abstract

• Myocardial herniation through a pericardial defect can mimic acute STEMI.
• Dynamic coronary compression in diastole suggests extrinsic vessel obstruction.
• Multimodality imaging is critical to diagnose congenital pericardial defects.
• Partial pericardial defects can cause myocardial strangulation or sudden death.
• Surgical repair of pericardial defects may prevent life-threatening complications.

## Graphical abstract

**Figure:**
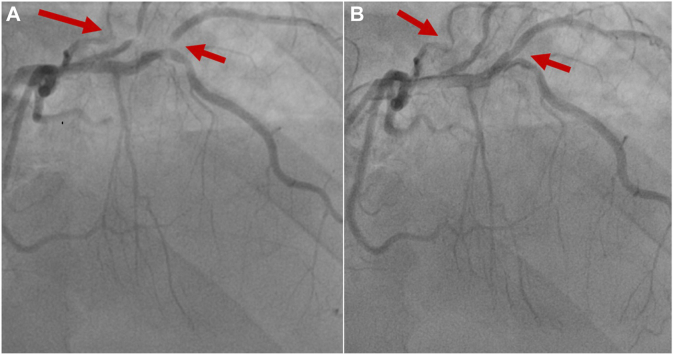

## Introduction

We describe the case of a 42-year-old man who presented with an ST-segment elevation myocardial infarction (STEMI) and was ultimately found to have external coronary compression from herniation of the left ventricle through a partial pericardial defect. Our case, diagnosed before surgery or autopsy, underscores the crucial role of multimodality imaging and highlights a key angiographic feature—dynamic coronary compression during diastole—that should alert interventional cardiologists to consider this diagnosis.

## Case Presentation

**Figure 1:**
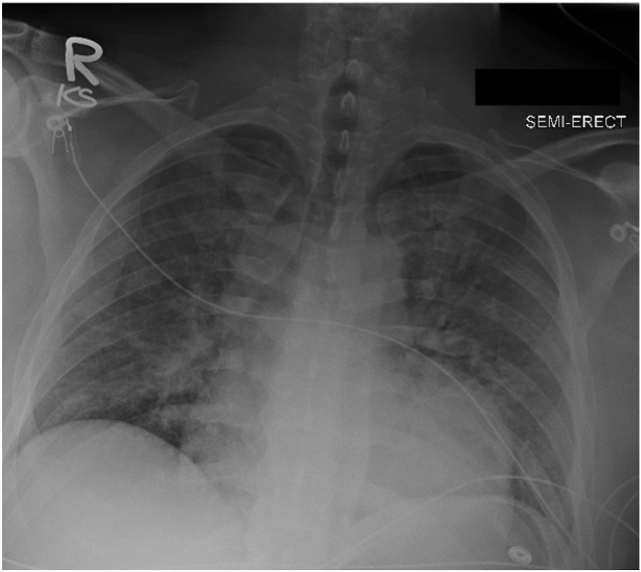
Single-view anteroposterior chest radiograph demonstrates diffuse bilaterial interstitial and alveolar opacities consistent with pulmonary edema.

A 42-year-old man awoke with sudden, substernal chest pain, worse with each heartbeat and associated with shortness of breath. Before this event, the patient was healthy aside from a non-COVID-19 viral illness 1 week before. There were no reported atherosclerotic risk factors, except a father with myocardial infarction at 60 years of age. Electrocardiography performed by emergency medical services raised concern for a lateral STEMI with 2-mm ST-segment elevation in leads I and aVL and reciprocal depressions in leads V_1_ to V_4_ (initial electrocardiogram not available). Chest radiography demonstrated diffuse bilateral interstitial and alveolar opacities consistent with pulmonary edema (Figure 1). Aspirin 325 mg was administered en route to a local emergency department. A ticagrelor load of 180 mg and 5000 unit heparin bolus were administered before emergent invasive coronary angiography (ICA) per STEMI protocol.

ICA revealed Thrombolysis in Myocardial Infarction grade 3 flow in all coronary vessels and dynamic compression of multiple coronary arteries. Extensive myocardial bridging or alternative pathology was considered. However, in the setting of a suspected lateral STEMI with corresponding hypokinesis on ventriculography, a drug-eluting stent (DES) was placed in the large first diagonal artery, the presumed culprit. Intervention to the remaining coronary arteries with apparent flow-limiting stenosis was deferred because of their small size and unclear etiology. Dual-antiplatelet therapy, a high-intensity statin, and a β-blocker were initiated. Transthoracic echocardiography (TTE) performed during this index hospitalization showed a preserved ejection fraction (EF) with lateral wall thickening, akinesis of the anterolateral myocardium, and hypokinesis of the inferolateral wall. Initial troponin I was elevated at 0.10 ng/mL (normal range, <0.04 ng/mL) and rose to 23 ng/mL, suggesting an acute myocardial infarction.

Despite percutaneous coronary intervention (PCI) with a DES, the patient had ongoing, intermittent, substernal chest pain, sharp in intensity and worse when lying on the left side. Treatment for suspected postinfarction pericarditis was initiated after a friction rub was appreciated on examination. The patient was discharged on postangiography day 4. The patient presented to our clinic 4 days after discharge for further evaluation of ongoing, intermittent chest pain.

**Figure 2:**
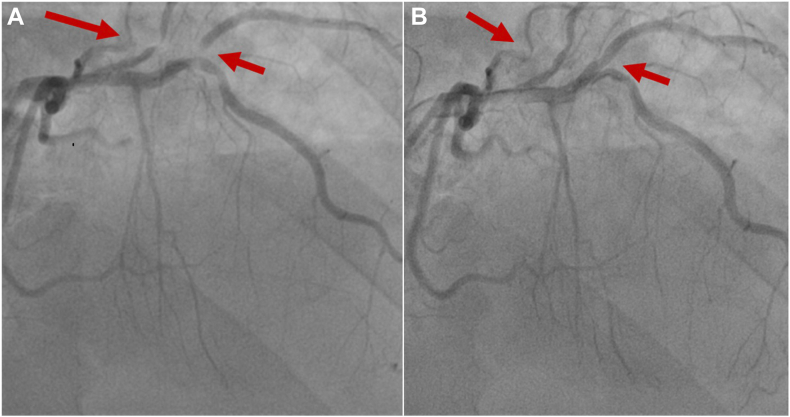
ICA, right anterior oblique cranial projection, demonstrates linear compression of coronary arteries during diastole **(A)** and normal-caliber coronary arteries with minimal filling defects in systole **(B)**.

**Figure 3:**
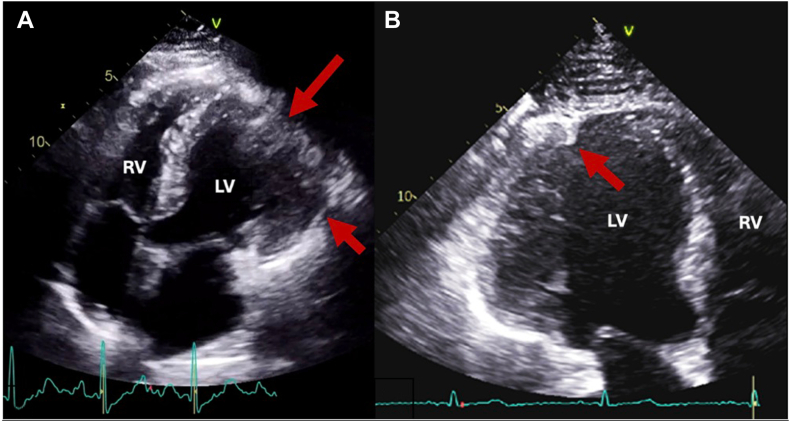
**(A)** Index hospitalization two-dimensional (2D) TTE, apical four-chamber view (left ventricle [LV] on *right*), demonstrates a thickened and poorly delineated anterolateral wall (*arrows*) in systole. **(B)** Repeat 2D TTE, zoomed-in apical four-chamber view (LV on *left*), demonstrates bulging of the thickened anterolateral ventricular wall beyond an area of constriction, where the myocardium appeared pinched. *RV*, Right ventricle.

Review of the outside ICA demonstrated cyclic linear compression of the first diagonal, ramus intermedius, and small first obtuse marginal arteries during diastole (Figure 2, Videos 1 and 2). Unlike systolic coronary compression seen in myocardial bridging, narrowing during diastole (ventricular filling) suggested coronary artery compression due to a fixed, external structure. On review of the outside echocardiogram, the lateral wall, though thickened and hypokinetic, was not well defined (Figure 3). The differential diagnosis expanded to include a pericardial or epicardial mass or cyst or herniation of the left ventricle through a pericardial defect.

Multimodality imaging was pursued to further evaluate the pericardium, myocardium, and surrounding structures. Repeat TTE showed a preserved EF (57%) with bulging of the lateral left ventricular (LV) wall beyond a point of constriction or crease, where the myocardium appeared pinched (Figure 3, Video 3). There was increased wall thickness and hypokinesis of the lateral and inferior segments, concerning for ischemia and edema.

**Figure 4:**
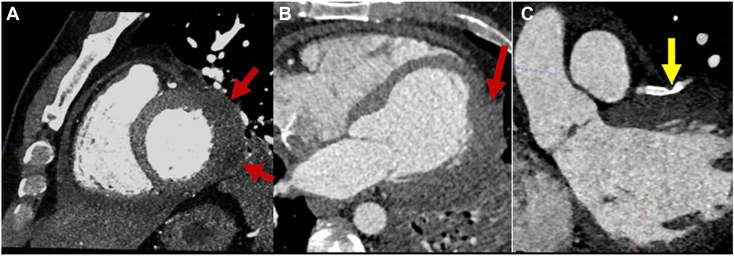
CCT, multiplanar reconstruction in sagittal **(A)**, axial **(B)**, and oblique coronal **(C)** views, demonstrates masslike thickening of the lateral, anterolateral, and inferolateral wall (*red arrows*) and severe focal stent deformation in the first diagonal artery (*yellow arrow*).

**Figure 5:**
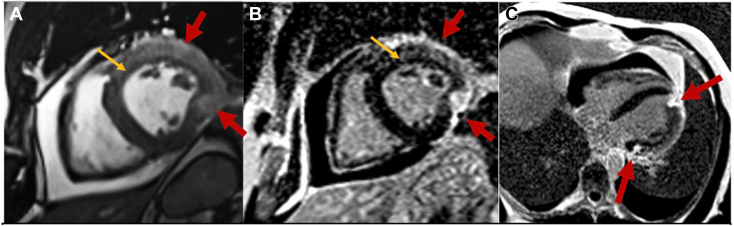
**(A)** CMR, T2-weighted short-tau inversion recovery short-axis image, demonstrates increased signal throughout the anterior and lateral walls (*red arrows*), sparing the subendocardium (*yellow arrow*), consistent with myocardial edema. **(B)** CMR, LGE sequence using phase-sensitive inversion recovery (PSIR), short-axis image, demonstrates subepicardial enhancement in the same distribution (*red arrows*) with subendocardial sparing (*yellow arrow*). **(C)** CMR, LGE sequence using PSIR, axial view, demonstrates thickening of the lateral wall of the left ventricle with more intense delayed enhancement along the lateral wall at the base and inferolateral mid and apical wall where the myocardium appears pinched, suggesting ring-shaped constriction with associated LGE.

Cardiac computed tomography (CCT) demonstrated abnormal masslike thickening of the lateral, anterolateral, and inferolateral LV wall segments, as well as severe focal stent deformation in the first diagonal artery (Figure 4, Video 4), suggesting compression of the coronary arteries by an epicardial structure. Cardiovascular magnetic resonance (CMR) showed ringlike constriction around a bulging lateral wall, with increased T2 signal and late gadolinium enhancement (LGE) with subendocardial sparing, suggesting edema and inflammation. The mid- and subepicardial LGE myocardial injury pattern was atypical for atherosclerotic coronary artery stenosis, which tends to affect the subendocardium first. A moderate anterior pericardial effusion was also appreciated (Figure 5, Videos 5 and 6), but the pericardium along the apex and lateral wall was not well seen. Together, these findings were concerning for herniation of an inflamed and edematous myocardium through an inflamed and deformed pericardium.

**Figure 6:**
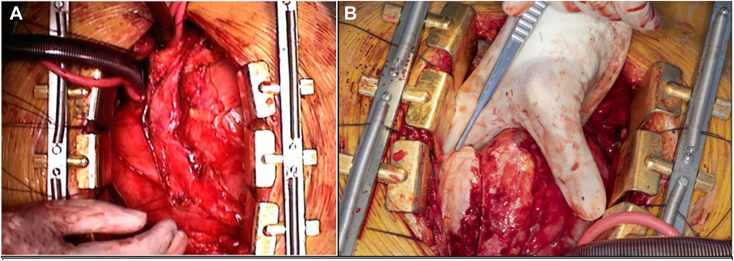
Surgical images at the start of pericardiectomy showing **(A)** an anterior partial pericardial defect and **(B)** white strangulated lateral myocardium with linear rim of restricting pericardium.

The patient was scheduled for pericardiectomy via median sternotomy with cardiopulmonary bypass 1 week after the diagnosis was made by multimodality imaging, approximately 1 month after their initial presentation with chest pain. Aspirin 81 mg daily was continued, and clopidogrel 75 mg daily (previously transitioned from ticagrelor because of dyspnea) was bridged perioperatively with intravenous cangrelor. Intraoperatively, the pericardium appeared thickened and inflamed, with dense adhesions to the anterior myocardium. A partial anterior pericardial defect was visualized (Figure 6) at the level of the left phrenic nerve, a surgical landmark of the anterolateral LV. The lateral LV wall appeared strangulated and friable (Figure 6), and all diagonal vessels were visibly indented and abutted by the pericardium. The anterior pericardium was resected and the pericardial edge mobilized to relieve coronary compression. The posterior defect was closed with polypropylene suture.

The patient was separated from cardiopulmonary bypass without difficulty, and postoperative recovery was uneventful. Clopidogrel 75 mg daily was resumed on postoperative day 3. After pericardiectomy, TTE demonstrated resolution of myocardial injury with a preserved EF of 55% and a mildly enlarged left ventricle with no regional wall motion abnormalities.

**Figure 7:**
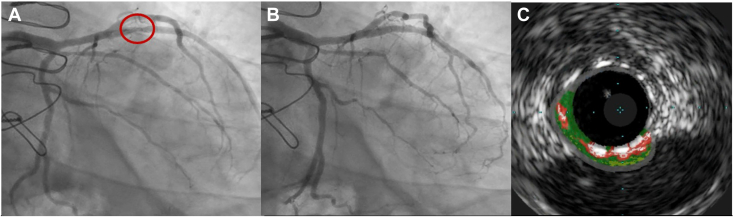
Postpericardiectomy ICA, right anterior oblique cranial projection, demonstrates stent deformation (**A**, *circle*) and results after balloon angioplasty of deformed D1 stent **(B)**. **(C)** Post-PCI virtual histology intravascular ultrasound display of the left anterior descending coronary artery demonstrates a well-expanded, well-apposed stent (*white*) with mild atherosclerotic plaque composed predominantly of fibrosis tissue (*green*) with minimal necrotic core (*red*).

Given the risk for in-stent restenosis and thrombosis due to the observed stent deformation, the patient underwent repeat percutaneous coronary balloon angioplasty before discharge (Figure 7, Video 7). Intravascular ultrasound ensured proper stent expansion and apposition to the vessel wall, with excellent angiographic results (Figure 7).

The patient completed 1 year of dual-antiplatelet therapy with aspirin and clopidogrel and has been doing well as of 24 months of follow-up.

## Discussion

Congenital pericardial agenesis is a rare condition, affecting fewer than one in 10,000 individuals, with a higher prevalence in men.^1^ It is characterized by partial or complete absence of the pericardium and results from defective development of the pleuropericardial membrane during the fourth to fifth weeks of embryogenesis.^1^ It may be found in isolation or associated with other congenital abnormalities.^1^

Complete absence of the pericardium, the most common form, is often asymptomatic and discovered incidentally.^1^ In contrast, partial pericardial agenesis (3%-5% of cases) is more likely to present with pleuritic, positional, or anginal chest pain or trepopnea, a pathognomonic symptom of dyspnea when lying on one side,^1^ as seen in our patient. Partial pericardial agenesis also carries a substantially higher risk for serious sequelae, including arrhythmias, coronary compression, sudden cardiac death, and myocardial herniation or strangulation.^1^ Thus, surgical correction (pericardiectomy, patch repair, or defect enlargement) is recommended for symptomatic partial pericardial defects to prevent such life-threatening complications.^1^

On the basis of our patient’s acute chest pain, ST-segment elevation on electrocardiography, elevated troponins, and myocardial edema on CMR, we suspect that our patient experienced acute herniation of the left ventricle through a previously silent pericardial defect, with resulting myocardial injury and coronary obstruction. In prior cases, herniation has occurred spontaneously^2^ or after a trigger.^3^, ^4^, ^5^ In our case, the recent viral illness and pericardial inflammation seen on CMR and intraoperatively suggest that pericarditis with worsening defect tightening could have predisposed to herniation. However, a spontaneous event without a trigger is also possible.

Our case is one of the few reported instances of ventricular herniation through a partial pericardial defect presenting with chest pain^2^, ^3^, ^4^, ^5^, ^6^, ^7^, ^8^, ^9^, ^10^, ^11^, ^12^, ^13^ and only the fourth to present as STEMI.^3^^,^^5^^,^^6^ Roughly half of prior cases were diagnosed intraoperatively during planned coronary bypass^9^^,^^10^^,^^13^ or postmortem.^5^^,^^6^ The others, including ours, were identified noninvasively with multimodality imaging,^2^, ^3^, ^4^^,^^7^^,^^11^^,^^12^ highlighting its importance. Among the rare STEMI presentations,^3^^,^^5^^,^^6^ only two patients, including ours, survived. Both were recognized with multimodality imaging and surgically corrected^3^; the two fatal cases were not diagnosed until autopsy, after fatal cardiogenic shock.^5^^,^^6^

**Figure 8:**
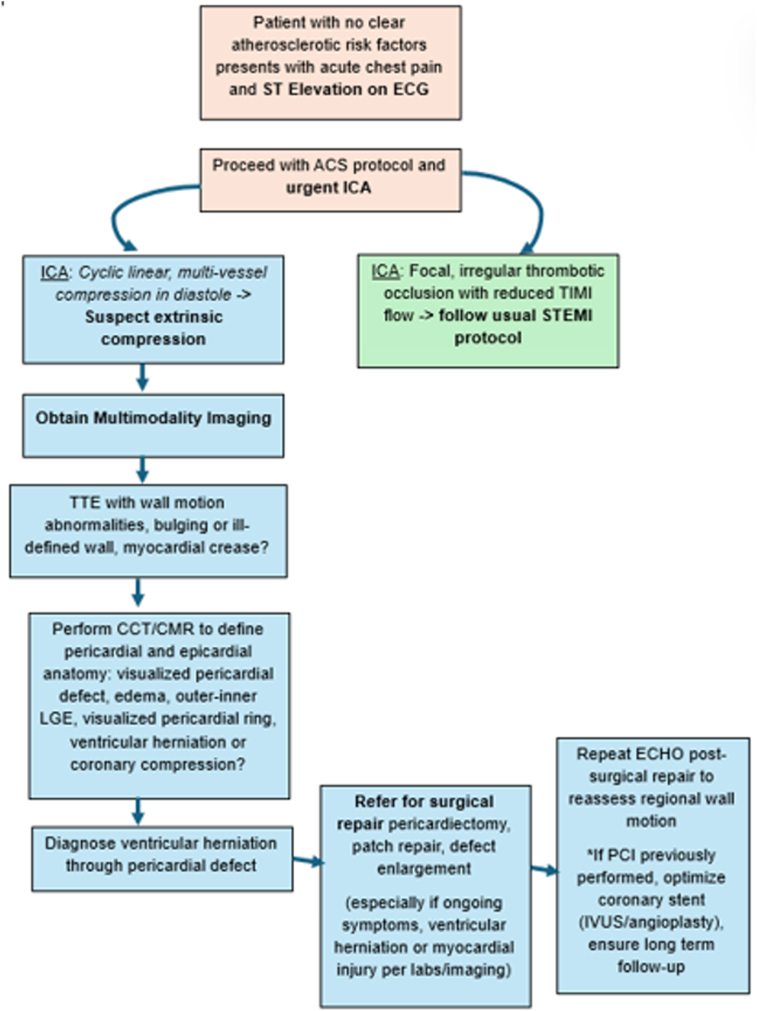
Proposed clinical algorithm for the diagnosis and management of ventricular herniation presenting as STEMI. *ACS*, Acute coronary syndrome; *ECG*, electrocardiography; *IVUS*, intravascular ultrasound; *TIMI*, Thrombolysis in Myocardial Infarction.

Because a lack of familiarity with this condition can delay diagnosis and definitive management, leading to increased morbidity and mortality, we propose an algorithm (Figure 8) for approaching these patients. When these patients present with ST-segment elevation on electrocardiography and acute chest pain, it is reasonable to transfer them to a PCI-capable hospital for urgent ICA per acute coronary syndrome guidelines.^14^ ICA provides the first clue that the ST-segment elevation and chest pain are not caused by acute plaque rupture. Cyclic compression of multiple coronary arteries in diastole^3^^,^^10^ is a telling angiographic finding that should raise suspicion for extrinsic coronary compression from a fixed pericardial rim or other epicardial structure and prompt further diagnostic evaluation.

Multimodality imaging is the crucial next step for confirming the diagnosis. Chest radiography in congenital pericardial agenesis may show marked leftward displacement of the cardiac silhouette and the classic “Snoopy sign”: an elongated, laterally displaced LV apex due to the absence of pericardial tethering.^2^ These findings were likely absent in our patient because the defect was partial rather than complete pericardial agenesis. No lung interposition was present on our patient’s radiograph.^1^

TTE can show indirect signs of pericardial agenesis, including paradoxical septal motion, more prominent right ventricle, excessive cardiac mobility, or abnormal acoustic windows due to cardiac displacement.^1^^,^^8^ In the setting of myocardial herniation through a pericardial defect and active myocardial ischemia, regional wall motion abnormalities can be present.^8^ Additionally, as seen in our case and another,^6^ the herniated wall may appear ill defined. A myocardial crease, in which myocardium appears pinched,^3^ is an excellent clue of overlying pericardial compression and herniation beyond the area of constriction.

CCT and CMR define pericardial anatomy with greater temporal and spatial resolution. On CCT, the pericardium is usually a 1- to 2-mm soft tissue structure lining the heart, best seen over the right atrium and right ventricle in both contrast and noncontrast images because of greater epicardial fat.^1^^,^^8^ Partial pericardial defects may be directly visualized as focal thinning or absence of the usual pericardial lining. Interposition of lung tissue between the aorta and pulmonary artery, a pathognomonic finding, is more often seen in complete left-sided pericardial defects.^1^^,^^8^ Per our case, ventricular herniation through a partial pericardial defect can cause myocardial edema and ischemia, with resulting myocardial thickening. Furthermore, CCT can define coronary anatomy. In our case, the kinking of a previously placed coronary stent on CCT provided indirect visualization of external coronary compression by the pericardial rim.

**Figure 9:**
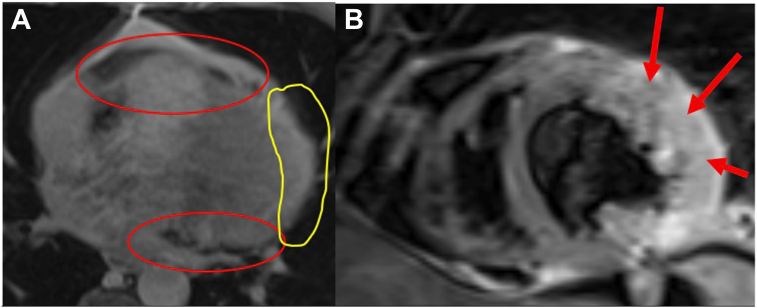
**(A)** CMR, axial postcontrast CAIPIRINHA (controlled aliasing in parallel imaging results in higher acceleration)–accelerated VIBE (volumetric interpolated breath-hold examination) with fat suppression in the delayed phase, demonstrates absence of the pericardium (*yellow oval*) along the lateral wall; remaining pericardium is visualized (*red ovals*). **(B)** CMR, short-axis T2-weighted triple inversion recovery sequence in our patient, demonstrates myocardial edema of the herniated wall (*red arrows*).

The reference standard for diagnosing pericardial defects is CMR because of its superior soft tissue resolution.^1^^,^^8^ The pericardium is often an intermediate- to low-signal structure on both T1- and T2-weighted imaging, enhanced by surrounding epicardial fat or pericardial fluid.^1^ In addition to corroborating findings on CCT, CMR clarifies the location and extent of the pericardial rim (Figure 9) and can demonstrate underlying myocardial edema,^1^ which appears as areas of high signal intensity on T2-weighted imaging (Figure 9). Prior cases have described myocardial and pericardial anatomy on CMR,^2^^,^^3^ but the LGE pattern in ventricular herniation is not well established.

In our case, LGE demonstrated a mid- and subepicardial pattern with subendocardial sparing. Although this pattern can be seen in myocarditis or certain nonischemic cardiomyopathies,^15^ its strict confinement to the herniated lateral wall, corresponding exactly to the area of mechanical impingement, supported a local mechanical rather than diffuse inflammatory process. Mechanistically, the rigid pericardial rim may have exerted repeated, external compression on the herniated myocardium, causing localized mechanical irritation and inflammation of the epicardial surface with extension into adjacent midmyocardial layers: an “outer-in” pattern. Although dynamic coronary compression was present, its transient nature may have permitted relative preservation of the subendocardium. This differs from traditional ischemia due to atherosclerotic coronary artery disease, in which the subendocardial tissue, the region most vulnerable to reduced perfusion, is affected first.^15^ Given limited prior descriptions of LGE findings in ventricular herniation through pericardial defects, however, our proposed mechanism remains speculative.

With heightened clinical suspicion based on the atypical coronary angiographic and multimodality imaging findings, we ultimately diagnosed our patient with herniation of the left ventricle through a congenital pericardial defect, allowing referral for pericardiectomy with clinical improvement. Whether stenting the diagonal artery and restoring some myocardial perfusion offered any mortality benefit is unclear. Patients presenting with unstable angina^4^ due to ventricular herniation have survived without immediate PCI and subsequently undergone delayed pericardial intervention. The other survivor with STEMI presentation^3^ did not appear to have undergone immediate ICA (the timing is unclear from the report) but was diagnosed early using multimodality imaging and underwent early pericardiectomy. Of the two cases presenting with STEMI with fatal outcomes, ICA was either delayed (8.5 hours after symptom onset)^6^ or not performed at all.^5^ The patient with delayed intervention survived 17 days on an intra-aortic balloon pump after DES implantation to the left anterior descending coronary artery but died once the balloon pump was removed. In the other case,^5^ a child living in the pre-PCI era presented late (the day following symptom onset), never underwent ICA, and died 19 hours after symptom onset from cardiogenic shock due to progressive strangulation. Given these results and our patient’s stent deformation, ongoing chest pain and CMR edema despite PCI, we suspect prognosis would have been poor without definitive surgical correction.

## Conclusion

Patients with partial congenital pericardial defects are at risk for complications, which include acute coronary syndromes and sudden cardiac death. In the setting of ventricular herniation and STEMI, ICA may provide the first diagnostic signs of pericardial agenesis, with dynamic compression of multiple coronary vessels in a linear pattern that occurs during diastole. A high clinical suspicion and multimodal imaging are important for definitive diagnosis and treatment planning. Patients with ventricular herniation through pericardial defects may require pericardiectomy for definitive management. The role of early PCI in these cases remains unclear.

## Ethics Statement

The authors declare that the work described has been carried out in accordance with The Code of Ethics of the World Medical Association (Declaration of Helsinki) for experiments involving humans.

## Consent Statement

Complete written informed consent was obtained from the patient (or appropriate parent, guardian, or power of attorney) for the publication of this study and accompanying images.

## Funding Statement

The authors declare that this report did not receive any specific grant from funding agencies in the public, commercial, or not-for-profit sectors.

## Disclosure Statement

The authors report no conflict of interest.

## Supplementary Data

Video 1 ICA, right anterior oblique cranial projection, demonstrates dynamic compression of multiple diagonal coronary arteries in diastole.

Video 2 ICA, right anterior oblique cranial projection, demonstrates marked dynamic compression of diagonal arteries in diastole.

Video 3 Repeat TTE, apical four-chamber view (left ventricle on *left*), demonstrates myocardial crease and bulging of lateral wall beyond area of constriction.

Video 4 CCT demonstrates thickening of lateral wall of the left ventricle.

Video 5 CMR, steady-state free procession short-axis sequence, demonstrates increased signal throughout the anterior and lateral walls (*red arrows*) sparing the subendocardium and associated hypokinesis.

Video 6 CMR, steady-state free procession four-chamber view, demonstrates lateral wall thickening and hypokinesis.

Video 7 Final ICA following pericardiectomy and balloon angioplasty of deformed D1 stent.
